# Listening to the elephant in the room: response-shift effects in clinical trials research

**DOI:** 10.1186/s41687-022-00510-6

**Published:** 2022-09-30

**Authors:** Carolyn E. Schwartz, I.-Chan Huang, Gudrun Rohde, Richard L. Skolasky

**Affiliations:** 1 DeltaQuest Foundation, Inc., 31 Mitchell Road, Concord, MA 01742 USA; 2 Departments of Medicine and Orthopaedic Surgery, Tufts University Medical School, Boston, MA USA; 3 Department of Epidemiology and Cancer Control, St. Jude Children’s Research Hospital, Memphis, TN USA; 4 Department of Clinical Research Sorlandet Hospital, Faculty of Health and Sport Sciences, University of Agder, Kristiansand, Norway; 5 Marie Curie Palliative Care Research Department, Division of Psychiatry, University College London, London, England; 6 Department of Orthopaedic Surgery, Johns Hopkins University School of Medicine, Baltimore, MD USA

**Keywords:** Response shift, Clinical trials, Scoping review, Outcomes

## Abstract

**Background:**

While a substantial body of work postulates that adaptation (response-shift effects) may serve to hide intervention benefits, much of the research was conducted in observational studies, not randomized-controlled trials. This scoping review identified all clinical trials that addressed response shift phenomena, and characterized how response-shift effects impacted trial findings.

**Methods:**

A scoping review was done of the medical literature from 1968 to 2021 using as keywords “response shift” and “clinical trial.” Articles were included if they were a clinical trial that explicitly examined response-shift effects; and excluded if they were not a clinical trial, a full report, or if response shift was mentioned only in the discussion. Clinical-trials papers were then reviewed and retained in the scoping review if they focused on randomized participants, showed clear examples of response shift, and used reliable and valid response-shift detection methods. A synthesis of review results further characterized the articles’ design characteristics, samples, interventions, statistical power, and impact of response-shift adjustment on treatment effect.

**Results:**

The search yielded 2148 unique references, 25 of which were randomized-controlled clinical trials that addressed response-shift effects; 17 of which were retained after applying exclusion criteria; 10 of which were adequately powered; and 7 of which revealed clinically-important response-shift effects that made the intervention look significantly better.

**Conclusions:**

These findings supported the presumption that response shift phenomena obfuscate treatment benefits, and revealed a greater intervention effect after integrating response-shift related changes. The formal consideration of response-shift effects in clinical trials research will thus not only improve estimation of treatment effects, but will also integrate the inherent healing process of treatments.

**Key points:**

- This scoping review supported the presumption that response shift phenomena obfuscate treatment benefits and revealed a greater intervention effect after integrating response-shift related changes.
- The formal consideration of response-shift effects in clinical trials research will not only improve estimation of treatment effects but will also integrate the inherent healing process of treatments.

## Introduction

Clinicians have long-acknowledged that patients adapt to their health condition [1]. They find ways to be happy despite restrictions in ambulation, respiration, and energy [2]. They find meaning and purpose even as their life narrows in scope or activity [3]. They re-think what is important to them [4], what “good quality of life” (QOL) means [5], what “moderate fatigue” means [6]. All of these underlying and often unspoken changes mean that, although the same person completes patient-reported outcomes repeatedly in a longitudinal study, they may be using different internal standards, referencing different values, or considering a different conceptualization of what the investigator is targeting [7–9]. These “response shifts” are critical to adaptation, and without response shift, patients’ ability to process the vicissitudes of life, health, and aging would be impaired [10, 11].

While a substantial body of work postulates that response-shift effects may serve to hide intervention benefits [7, 8, 12, 13], much of the research concerns observational studies that are not randomized controlled designs. Response-shift effects are, however, likely of relevance to clinical-trials research. The intervention and control/placebo groups likely adapt differently, particularly if the intervention is effective. If they adapt differently, what are the implications for the treatment’s observed benefit? Does response shift play a role in non-inferiority trials? What can be learned in such trials if, for example, treatment arm differences are negligible? Is response shift ignorable by clinical trialists? By governmental agencies responsible for vetting new drugs?

We postulate that clinical trialists have largely ignored response shift phenomena in pivotal trials, and that any such investigations would be secondary analyses. We believe that this context may relate to concerns that response-shift studies will undermine pivotal trial findings, no matter how well-designed the control group. By ignoring this widely acknowledged human-adaptation process, however, trialists are co-existing with an “elephant in the room.” In other words, everyone knows it’s there and no one is talking about it.

The present work implemented a scoping review of the literature to find all clinical trials that addressed response shift phenomena, and to characterize how response-shift effects impacted trial findings. Such a review is an approach for evidence synthesis [14] that seeks to identify knowledge gaps, clarify concepts, or investigate research conduct [14]. A scoping review may be a precursor to a systematic review, the latter seeking to uncover and appraise international evidence, utilizing methods that minimize bias using rigorous methods to synthesize information related to a particular question, and to inform practice [14]. Accordingly, and consistent with a scoping-review approach [15], the present work seeks to describe the clinical-trials literature with regard to response-shift investigations. It does not seek to quantify average effect sizes, which would be more appropriate to a meta-analysis (e.g., [16]).

## Methods

### Data sources and search strategy

We implemented a scoping review of the medical literature from 1968 to 2021. The goal of the scoping review was to focus on response-shift research in the context of clinical trials. Using the search terms (i.e., keywords) “response shift” and “clinical trial,” we searched the following databases: Pubmed, CINAHL Plus, Embase, PsycInfo, and Google Scholar. We then combined the search results and removed any duplicate literature.

### Article selection and characterization

The research team met repeatedly in advance of the initial screen and selection of articles to make sure we all understood the screening methodology. This approach was similar to an earlier project done by this research team, in which we demonstrated that this collaborative approach was efficient and achieved a high level of reliability [11]. The first author (CES) then examined the list of search results, and identified articles that explicitly examined response-shift effects in clinical-trials data. Articles were included at this initial stage if they were a clinical trial that explicitly examined response-shift effects. Articles were excluded at this initial stage for the following possible reasons: were not a clinical trial (e.g., observational study, literature review, protocol only; were an abstract only, not a full report); or if response shift was mentioned only in the introduction section and/or discussion section of the paper.

The resulting set of clinical-trials articles were then divided among four raters (CES, ICH, GR, RS) for further characterization of: (a) the main research question; (b) the drug or intervention being evaluated; (c) the clinical-trial design; (d) patient population; (e) patient-reported outcome tools used; (f) response-shift methods used; (g) whether there were hypotheses regarding response-shift effects and (h) response-shift findings. At this stage, further exclusions were made if the article focused only on non-randomized study participants or was not a clear example of response shift. Additionally, we excluded articles that used the then-test method, due to the plethora of studies documenting problems of reliability and validity of this obsolete method [17–19]. All summary information about the final set of included articles was double-checked by all four raters to ensure accuracy of presented syntheses.

### Synthesis of review results

We grouped the final set of retained articles by: (a) whether the study was a primary or secondary/post-hoc analysis; (b) whether the intervention was drug/medical device/surgery or psychosocial/behavioral/nursing intervention; and (c) whether the stated focus of the work was primarily methodological or on the clinical impact of response-shift effects.

We then examined whether response shift affected trial results as a function of the study’s statistical power for the specific response-shift detection method used, using well-accepted guidelines for statistical-power considerations [20–22]. For example, using Cohen’s criteria for a comparison of means, a study would need to have at least 26 people per group or treatment arm to be powered to detect a large effect size [21]. For multivariate analytic methods that require larger sample sizes to yield robust estimates, larger sample sizes would be needed [23]. For example, a rule of thumb for structural equation modeling is 200 people per group/time point being evaluated, which would enable adequate power robust estimates of loadings of 0.90 but not for loadings of 0.80 [24]. Although some recent studies indicate a range of sample size goals for such modeling, the differences in sample sizes are primarily driven by number of variables in the model. In studies investigating response-shift effects as measurement invariance using a structural equation modeling framework, we believe the general rule of thumb is a reasonable criterion. Ideally, studies would be powered to detect at least a medium effect size since this corresponds to clinically significant change [25]. Being powered to detect small effect sizes would correspond to current estimates of response-shift effects in observational research [16], although a clinical trial of a potent intervention might be expected to yield at least medium response-shift effects. For the purposes of the current work, “adequate” was defined to be 80% power, α = 0.05, to detect at least a large effect size given the specific statistical method used. The point estimate for small, medium, and large effect sizes differs depending on the statistical method used, and the interested reader is referred to Cohen’s seminal paper for examples using common behavioral-science methods [21].

If adequately powered, we evaluated whether the response-shift adjustment made the treatment look more or less effective. In other words, did the treatment(s) being assessed in the clinical trial have a more or less beneficial impact on outcomes when response-shift effects were considered?

## Results

### Descriptive characteristics of included articles

**Fig. 1:**
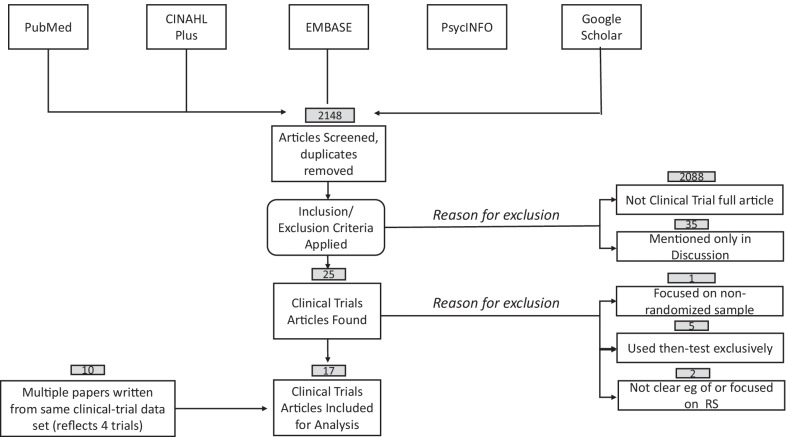
Flow chart of the article selection process for final set of retained articles

The database search yielded 2148 unique references, 116 of which were found using PubMed, CINAHL Plus, EMBASE, and PsycINFO, all of which were duplicated in the Google Scholar search which yielded 2148 articles. After excluding ineligible articles (2088 not clinical trials, 35 mentioning response shift only in introduction or discussion), a set of 25 articles was included in the scoping review (Appendix Table 2) (Fig. 1). Further exclusion was done because the article focused on a non-randomized sample (n = 1) [26]; the article used the then-test exclusively (n = 5) [27–31]; or because the article was not a clear example of response shift (n = 2) [32, 33] (Table 1, Fig. 1). The remaining 17 articles included ten papers reflecting four trials, where 11 of which addressed a distinct response-shift hypothesis (Fig. 1).

**Fig. 2:**
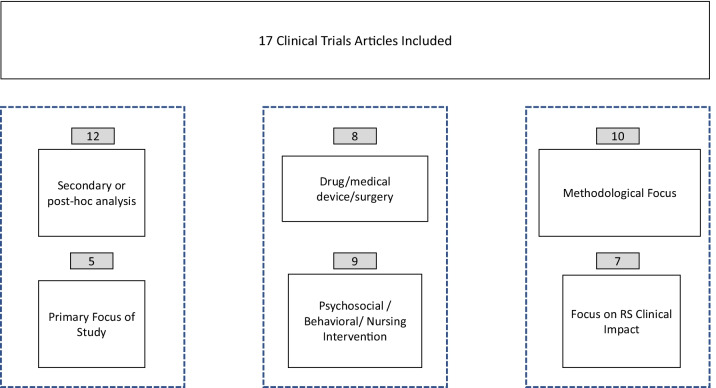
Characterization of clinical-trials articles included

The 17 articles were predominantly secondary or post-hoc analyses (n = 12) [34–45], rather than explicitly designing the study to address response-shift effects (n = 5) [19, 46–49] (Fig. 2). Eight of the retained articles addressed drug, medical-device, or surgical interventions [11, 36, 37, 39, 40, 43, 44, 48], and nine addressed psychosocial, behavioral, or nursing interventions [34, 35, 38, 41, 45–47, 49, 50]. Ten of the articles were focused on methodological development [19, 34–38, 40, 41, 50, 51], and seven on the clinical impact of response shift [11, 39, 43–46, 49].

### Substantive findings

**Fig. 3:**
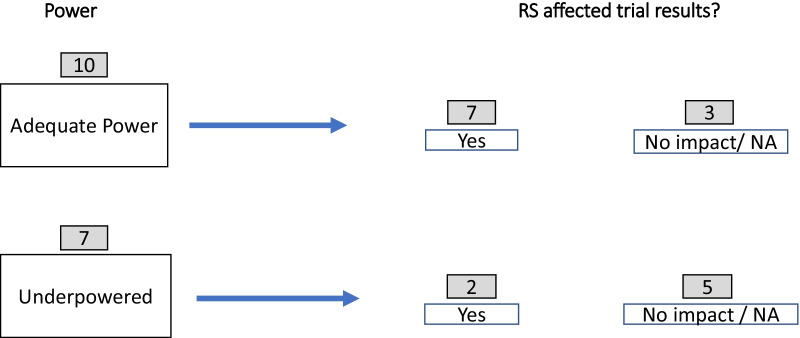
Impact of power on response-shift effects on trial results

Of note, the articles documented that response shift affected trial results more often than not. This impact was also associated with the statistical power of the comparisons done (Fig. 3). Among the 10 retained articles that had adequate power, seven documented a clinically-important response-shift effect that affected trial results [11, 37, 38, 43, 44, 46, 49], two did not [47, 50], and one did not address the clinical impact of response shift [41]. Among the seven retained articles with inadequate power, two documented a clinically-important response-shift effect (one better [39], one worse [35]), and five documented no impact on the estimated intervention impact [36, 40, 45, 47, 48].

**Fig. 4:**
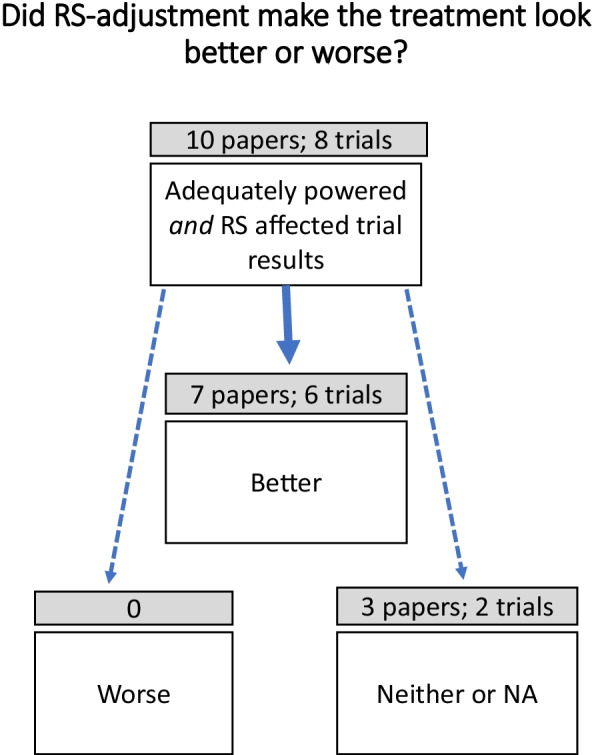
Impact of response-shift adjustment on estimated treatment benefit

Considering only the ten adequately powered studies that documented a response-shift impact on the intervention, seven revealed a clinically-important response-shift effect that made the intervention look significantly better [11, 37, 38, 43, 44, 46, 49], none made it look worse, and three documented no impact [34, 50], and one did not address the impact on the intervention effect [41] (Fig. 4). These findings support the long-standing presumption that response shift phenomena may serve to obfuscate treatment benefits [7–10, 52, 53].

The studies that revealed a greater intervention effect after integrating response-shift related changes. Consistent with intervention goals, there were changes in priorities/preferences after Advance Care Planning interventions [46, 49]; changes in conceptualization after post-stroke rehabilitation [34]; changes in internal standards for physical functioning after anti-hypertensive treatment [37]; and changes in internal standards resulting in increased honesty about risky drinking behavior after a motivational-interviewing intervention [38]. Larger differences in mental-health functioning were found between treatment arms, after considering response-shift effects in a trial comparing a highly effective drug to placebo for patients with a chronic progressive neurological disease [11, 43]; and two effective treatments in a non-inferiority trial for a chronic blood disorder were found to yield “better than normal” QOL compared to the general population [44].

**Table 1:** Clinical Trials Articles Found After Initial Inclusion/Exclusion Criteria Applied

References	Secondary analysis?	Retained or excluded (reason for exclusion)	Methodological or clinical focus?	Drug/medical/surgery or Psychosocial/Behavioral/Nursing Intervention?	Sample overlap with another paper?	Power adequate if retained?	Response shift made treatment look better? Worse? Neither?
Schwartz and Sendor [26]	Yes	Excluded (focused on non-randomized sample)	Clinical	Psychosocial/Behavioral/Nursing	No	NA	NA
Bernhard et al. [27]	Yes	Excluded (used then-test exclusively)	Clinical	Drug/medical/surgery	Yes, references [27, 28, 31]	NA	NA
Bernhard et al. [28]	Yes	Excluded (used then-test exclusively)	Clinical	Drug/medical/surgery	Yes, references [27, 28, 31]	NA	NA
Schwartz et al. [46]	No	Retained	Clinical	Psychosocial/Behavioral/Nursing	No	Yes	Better
Bernhard et al. [31]	Yes	Excluded (used then-test exclusively)	Clinical	Drug/medical/surgery	Yes, references [27, 28, 31]	NA	NA
Ahmed et al. [29]	No	Excluded (used then-test exclusively)	Methodological	Psychosocial/Behavioral/Nursing	Yes, references [19, 29, 47]	NA	NA
Ring et al. [30]	Yes	Excluded (used then-test exclusively)	Clinical	Drug/medical/surgery	No	NA	NA
Ahmed et al. [47]	No	Retained	Methodological	Psychosocial/Behavioral/Nursing	Yes, references [19, 29, 47]	No	Neither
Ahmed et al. [50]	No	Retained	Methodological	Psychosocial/Behavioral/Nursing	Yes, references [19, 29, 47]	Yes	Neither
Mayo et al. [34]	Yes	Retained	Methodological	Psychosocial/Behavioral/Nursing	Yes, references [32, 34]	Yes	Neither
Ahmed et al. [35]	Yes	Retained	Methodological	Psychosocial/Behavioral/Nursing	No	No	Worse
Robertson et al. [36]	Yes	Retained	Methodological	Drug/medical/surgery	No	No	NA
Mayo and Scott [32]	Yes	Excluded (not clear example of response shift)	Clinical	Psychosocial/Behavioral/Nursing	Yes, references [32, 34]	NA	NA
Gandhi et al. [37]	Yes	Retained	Methodological	Drug/medical/surgery	No	Yes	Better
Nirenberg et al. [38]	Yes	Retained	Methodological	Psychosocial/Behavioral/Nursing	No	Yes	Better
Sajobi et al. [39]	Yes	Retained	Clinical	Drug/medical/surgery	No	No	Better
Mollerup and Johansen [33]	Yes	Excluded (not clear example of response shift)	Methodological	Psychosocial/Behavioral/Nursing	No	NA	NA
Machuca et al. [48]	No	Retained	Methodological	Drug/medical/surgery	No	No	Neither
Hoerger et al. [49]	No	Retained	Clinical	Psychosocial/Behavioral/Nursing	No	Yes	Better
Murata et al. [40]	Yes	Retained	Methodological	Drug/medical/surgery	No	No	Neither
Sanders et al. [41]	Yes	Retained	Methodological	Psychosocial/Behavioral/Nursing	No	Yes	NA
Schwartz et al. [43]	Yes	Retained	Clinical	Drug/medical/surgery	Yes, references [11, 43]	Yes	Better
Schwartz et al. [11]	Yes	Retained	Clinical	Drug/medical/surgery	Yes, references [11, 43]	Yes	Better
Verdam et al. [45]	Yes	Retained	Clinical	Psychosocial/Behavioral/Nursing	No	No	Neither
Schwartz et al. [44]	Yes	Retained	Clinical	Drug/medical/surgery	No	Yes	Better

## Discussion

The present scoping review documents an emerging literature on response shift in randomized-controlled clinical-trials research. This literature draws on medical and behavioral interventions, and focuses on a both methodological and clinical-impact studies. Among the two-thirds with adequate statistical power for the response-shift analyses implemented, it was eminently clear that response shift phenomena affected trial results and predominantly in the direction of revealing more substantial treatment benefits. Most of the studies suggesting no impact of response shift phenomena were underpowered for the response-shift analyses implemented, thereby undermining their conclusions. Thus, when the “elephant” was empowered to “speak”, the response-shift effects detected in adequately powered studies suggested greater treatment benefits than previously found in the pivotal trials.

The implications of the present work are substantial for clinical trialists. First, response-shift effects are likely important for better understanding treatment effects for both medical and behavioral interventions. Further, this better understanding is unlikely to denigrate the benefit of the treatment effects, as estimated by analyses that do not explicitly consider response-shift effects. For example, clinical trials that do not document a positive impact on mental health in the context of a powerful treatment that modifies disease progression, may well be “hiding” a response-shift effect that belies the greater benefit of the drug (e.g., [11, 43]). Uncovering such effects is an important and clinically relevant outcome.

A second implication is that clinical trialists interested in evaluating response-shift effects in their trial data should pay attention to statistical power considerations when selecting the response-shift detection method. Our study suggests that being underpowered studies were more likely to conclude that response shift phenomena did not affect trial results. Recent developments in response-shift methods provide efficient and effective ways to examine response-shift effects even in the context of small samples [11, 43] or non-inferiority trials [44]. These include adaptations of random-effects modeling [54], equating [55], and case–control studies [56].

The present study also has implications for the Federal Drug Administration (FDA) and European Medicine Agency (EMA) in their process for considering new drug applications. If considering response-shift effects leads to an increased estimate of the treatments effect, then it should be standard practice to use methods that integrate response-shift effects in clinical trials analyses. Of note, such methods should have a strong evidence base for reliability and validity, thus specifically excluding use of the then-test method. Additionally, treatments that enable response-shift effects are likely more desirable than those that do not. While this idea is apparent in the context of rehabilitative nursing interventions (e.g., [34]), it is also desirable in other contexts. A drug with severe toxicities may make it difficult for the patient to reprioritize or reconceptualize QOL, particularly in the control arm if the drug is the current standard of care, because so much of the patient’s time is spent in suffering. Explicitly requiring analyses that consider response-shift effects for new drug applications would be an appropriate policy implication of the present work. Further, trialists should be encouraged to evaluate response effects separately for the interventional arm and control arm. If the response-shift effect is detected, then the treatment findings should further incorporate or adjust the response-shift effects. Accordingly, the FDA and EMA should integrate response-shift effects into their guidelines and operational standards for incorporating response-shift evaluation in the future trials, particularly with regard to considerations of statistical power for the response-shift detection method(s) being used.

The present work had notable advantages, such as considering a large set of potential articles and reducing the set for further consideration based on clear and replicable criteria. It is possible, however, that this large set was limited by publication bias, that is that null results were not deemed publishable and thus not available for inclusion. This source of bias may have distorted the findings of the present work.

## Conclusions

In summary, this scoping review identified 25 randomized-controlled clinical trials that addressed response-shift effects. A subset of 17 were retained after implementing exclusion criteria, of which 10 were adequately powered to implement the statistical methodology used. These papers generally documented a larger treatment effect after considering response-shift effects. This work thus demonstrated that response shift has an effect on clinical-trial outcomes, and supports the recommendation that current and future researchers should incorporate methods to detect response shift when reporting results, especially when reporting null results. The formal consideration of response-shift effects in clinical trials research will thus not only improve estimation of treatment effects, but will also integrate the inherent healing process of treatments. Adaptation is part of a positive outcome process, and thus should be central to clinical trials analyses.

## Data Availability

The data used in these analyses are publicly-available publications. Requests for summary tables may be shared upon review.

## Appendix

See Table 2.

**Table 2:** Clinical trials articles found after initial inclusion/exclusion criteria applied

References	Characteristics
Schwartz and Sendor [26]	Main Research Question: Explores the impact of helping others on the physical and psychosocial wellbeing of the provider Drug/Intervention: Non-directive telephone support to patients with multiple sclerosis Type of Clinical Trial: Secondary analysis of non-randomized providers of one treatment arm from RCT RS Hypotheses: Peer support may be a benefit to the helper through changing self-evaluation despite no change in objective function or circumstances Sample: Lay peer support providers (n = 5) of patients with multiple sclerosis who were trained as part of a clinical trial to evaluate the role of peer support; 132 patients with MS who were randomized either to directive or non-directive group intervention. Telephone group n = 67, coping group n = 60, non-randomized peer supporters who implemented the telephone group n = 5 RS Methods: Effect sizes were estimated for each QoL outcome in each group; Chi-square test of association between small, medium, and large effect sizes RS Findings: Peer supporters had 3–8 times the benefit on a number of measures (psychosocial role performance, adaptability, well-being) compared to patients receiving an intervention
Bernhard et al. [27]	Main Research Question: To evaluate whether patients with colon cancer undergoing surgery with or without adjuvant chemotherapy change the internal standards on which they base their QL estimation, and, if they do so, whether this reframing alters interpretation of QL findings Drug/Intervention: Post-operative adjuvant chemotherapy, two arms received different dose as compared to observation only Type of Clinical Trial: A phase-III trial (the Swiss Group for Clinical Cancer Research [SAKK] 40/93) for colon cancer RS Hypotheses: Expected that patients’ estimates of their pre-surgery QL would be lower after surgery compared to before. Also, expected that the patients' retrospective estimates of their QL at the beginning of adjuvant therapy would be lower than estimates made beforehand. This reframing was expected to be stronger in patients receiving chemotherapy compared to those without Sample: 187 patients with early colon cancer (no. per arm not provided) RS Methods: Then-test method. The first hypothesis (i.e., worse scores of retrospective estimations) was investigated by paired t-tests between the pre- and then-test assessments separately for surgery and the adjuvant phase, the latter separately by treatment and overall. The second hypothesis (i.e., worse scores of retrospective estimations in patients with chemotherapy as compared to those without) was investigated by F-tests of changes between pre- and then-tests RS Findings: (1) Patients reported their pre-surgery QL after surgery significantly lower than before the surgery, and their pre-adjuvant QL under treatment or observation also lower than at the beginning. (2) In the adjuvant phase, in contradiction to our hypothesis, chemotherapy had almost no impact on these changes attributed to reframing. However, conventionally assessed changes indicated an improvement in QL. (3) After adjustment of current QL scores under treatment or observation to patients' retrospective estimation, the treatment effects were diluted but the overall improvement was substantially amplified in most QL indicators
Bernhard et al. [28]	Main Research Question: To evaluate whether the response shift affects the perception of health for utility evaluation in cancer clinical trials Drug/Intervention: Post-operative adjuvant chemotherapy, two arms received different dose as compared to observation only Type of Clinical Trial: A phase-III trial (the Swiss Group for Clinical Cancer Research [SAKK] 40/93) for colon cancer RS Hypotheses: Expected that patients' estimates of their pre-surgery QL would be lower after surgery compared to before. Also, expected that the patients' retrospective estimates of their QL at the beginning of adjuvant therapy would be lower than estimates made beforehand. This reframing was expected to be stronger in patients receiving chemotherapy compared to those without Sample: 187 patients with early colon cancer (no. per arm not provided) RS Methods: Then-test method RS Findings: Patients estimated pre-surgery health was worse after surgery than before (*P* = 0.01), and their estimated pre-adjuvant health was worse under treatment or observation than at the beginning (*P* = 0.001), in agreement with QL indicators. Chemotherapy had no impact on these changes attributed to a response shift. Conventionally assessed changes between the beginning of adjuvant treatment or observation and 2 months later indicated no change in subjective health. Change scores relative to patients’ retrospective estimation revealed an improvement (*P* = 0.004) in this period
Schwartz et al. [46]	Main Research Question: The primary aim was to evaluate the importance of the facilitated interview portion of the La Crosse intervention in a prospective RCT. A secondary aim was to examine whether the La Crosse intervention affected patients’ preferences by education the patient about the benefits and burdens of end-of-life Drug/Intervention: Advanced Care Planning intervention. Control group received usually care Type of Clinical Trial: Single blind RCT RS Hypotheses: The facilitated interview used in the La Crosse intervention is effective in improving short-term outcomes associated with end-of-life care Sample: 61 Ambulatory geriatric patients. Respecting choices interview n = 30, nondirective interview n = 30 RS Methods: RS in patients QOL conceptualization were determined using t-test comparing a within-subjects congruence score of the Beliefs and Value Questionnaire. RS of in internal standards were assessed using within-group t-tests on the difference scores of the VAS item s for pain, anxiety, and alertness. The difference in then-test values and baseline values estimated changes in internal standards RS Findings: The intervention group became less willing to tolerate poor health status, suggesting RS in conceptualization of QOL. There were no changes during follow-up on VAS items for pain, anxiety, or alertness suggesting the participants in both arms did not make response shifts in internal standards. Thus, the patients did not seem to recalibrate their standards of QOL outcomes but rather to reflect on and define their own values after having a better understanding of benefits and burdens of end-of-life care
Bernhard et al. [31]	Main Research Question: To evaluate whether the meaning of QL remains constant over time among patients with early colon cancer Drug/Intervention: Post-operative adjuvant chemotherapy, two arms received different dose as compared to observation only Type of Clinical Trial: A phase-III trial (the Swiss Group for Clinical Cancer Research [SAKK] 40/93) for colon cancer RS Hypotheses: The meaning of QL for patients would be changed across different phases of disease and treatment Sample: 186 patients with early colon cancer (no. per arm not provided) RS Methods: Then-test method RS Findings: Response shift effect was analyzed based on the surgery pre-test and then-test, and the adjuvant pre-test and then-test. There was no evidence to suggest that the parameters for all six QL domains changed
Ahmed et al. [29]	Main Research Question: To assess whether the recovery process following stroke altered individuals’ perceptions of past health status and the impact that change in internal standards (RS) had on ratings over time Drug/Intervention: Home intervention on stroke outcome Type of Clinical Trial: 146 stroke patients and 50-control (family caregivers of the patients) study RS Hypotheses: 1) That individuals with stroke would experience changes in internal standards, not experienced by the control group, 2) given the expected improvements in physical health, individuals with stroke would, on average, retrospectively report worse baseline health status (report lower then test ratings), when rated retrospectively, at 6 weeks and 24 weeks poststroke than what they originally reported it to be at the previous evaluation, 3) that as time goes on and people’s functional ability improves, that individuals would retrospectively reevaluate how they were in the past, 4) those who improved on the objective measures would also have greater improvements in RS adjusted change compared to those who deteriorated or did not change Sample: 146 stroke patients and 50 controls RS Methods: The then-test RS Findings: The pattern of mean scores was indicative of changes in internal standards among individuals with stroke but not for the control group. Memory had an impact on estimates of response shift. Hypotheses related to the objective criterion measures were not supported
Ring et al. [30]	Main Research Question: To determine whether response shift would influence the measurement of treatment efficacy in edentulous patients using individualized quality of life measure (the SEIQOL) through a then-test Drug/Intervention: Osseo-integrated implant supported dentures vs. conventional dentures within edentulous patients Type of Clinical Trial: The first phase of a randomized controlled clinical trial designed to assess the impact on QOL of implant supported dentures compared with high quality conventional dentures. Patients wore new high quality conventional dentures for three months before being randomized either to continue with the conventional dentures or to receive implants RS Hypotheses: High quality dentures or osseo-integrated implants was likely to result in improved eating, communication, appearance, social life, and individual QOL (IQOL). Since the treatment periods were long (three months and six months), the psychological impact of treatment was likely to be marked on the main outcome measure individualized QOL Sample: 117 edentulous patients (no. per arm not provided) RS Methods: Then-test method (baseline vs. three months) RS Findings: Unadjusted SEIQOL index scores revealed no significant impact of treatment at three months (p = 0.33). However, the then-test at 3 months revealed that patients retrospectively rated their baseline IQOL as significantly lower (P < .001) than they had rated it at the time (then-test baseline). Comparison of the 3 month scores with this readjusted baseline indicated a significant treatment effect (then-test baseline, p = 0.016). Also 81% of patients nominated at least one different IQOL domain at 3 months
Ahmed et al. [47]	Main Research Question: To contrast three methodologic approaches for evaluating RS to develop a proposed set of HRQL measurement recommendations under circumstances where RS is expected to occur Drug/Intervention: Home intervention on stroke outcome Type of Clinical Trial: 150 stroke patients and 50 -control (family caregivers of the patients) study RS Hypotheses: None Sample: 150 stroke patients of whom 92 completed the Patient Generated Index RS Methods: Structural Equation Modeling, Then-test and an individualized approach RS Findings: The Structural Equation Modeling did not show a response shift, contrary to the results of the then test and the individualized approaches
Ahmed et al. [50]	Main Research Question: To assess reconceptualization of HRQL and change of individual values among persons with stroke during the first six months of recovery Drug/Intervention: Home intervention on stroke outcome Type of Clinical Trial: Clinical trial Patients—completers (n = 92) and non-completers (n = 58) of Patient Generated Index RS Hypotheses: None Sample: 146 stroke patients and 50 controls RS Methods: Interviews using Patient Generated Index RS Findings: After reviewing the 46 interviews, 13 (28%) people were classified as having expressed verbalizations reflecting a RS. Many individuals (n = 31) who did not verbalize a response shift had changes in the areas selected between both evaluations
Mayo et al. [34]	Main Research Question: Do different RS detection methods lead to different conclusions about the existence of RS? Drug/Intervention: 6 weeks of need based nursing interventions delivered by a nurse caregiver following hospital discharge Type of Clinical Trial: Secondary analysis of stratified, balanced, randomized clinical trial; no blinding RS Hypotheses: Difference methods of RS detection lead to different conclusions about its presence Sample: 150/190 persons returning home directly from acute-care hospital following first/recurrent stroke with need for health-care supervision post-discharge due to low function, comorbidity, or isolation; 126 (63 in each group) had the Then-Test RS Methods: Then-test for RS at the individual level; EFA/CFA of SF-36 and stepwise linear regression for RS at the group level; Analysis of residuals from predictive model of EQ-VAS over time RS Findings: Residual analysis revealed RS in half the sample. Most of the response shift was positive, people assessed their health better than what would be predicted by their stroke-related disabilities alone; this is a desired feature of the rehabilitation process
Ahmed et al. [35]	Main Research Question: 1) To evaluate whether persons with chronic obstructive pulmonary disease (COPD) experience a response shift after participating in a self-management program. 2) To compare the Oort and Schmitt Structural Equation Modeling approaches Drug/Intervention: 4 weeks of hospital-based self-management education, after which they were randomized to 8 weeks of either home-based or outpatient hospital-based rehabilitation Type of Clinical Trial: Unblinded 2-arm trial RS Hypotheses: None Sample: 252 patients with COPD: outpatient-based rehabilitation n = 126, home-based rehabilitation n = 126 RS Methods: The Oort and Schmitt Structural Equation Modeling methods RS Findings: The Oort approach showed significant changes between the no-RS model and models removing invariance constraints for the residual of the CRQ dyspnea (uniform recalibration) and intercepts of the SGRQ activity and impact subscales (nonuniform recalibration). Change in factor means showed changes in the physical health factor, which was slightly lower in unadjusted as compared with the RS adjusted model. The Schmitt procedure was not supportive of any RS effect
Robertson et al. [36]	Main Research Question: To understand what QOL means to people and how this influences the interpretation of HRQOL questionnaires Drug/Intervention: Intensive bisphosphonate therapy vs symptomatic therapy Type of Clinical Trial: multicenter RCT RS Hypotheses: Reference frame choice changes over time Sample: Paget's Disease: PRISM RCT with n = 1331, of whom 21 were interviewed to assess reference frame RS Methods: Cognitive interviewing RS Findings: Health not the most important factor in evaluating QOL. Social relationships were impacted by reduced vitality. Reference frames changed over time and interacted in complex ways, which may create potential bias when supplemental questions are not used to assess standards of comparison
Mayo and Scott [32]	Main Research Question: Drug/Intervention: 6 weeks of need-based nursing interventions delivered by a nurse caregiver following hospital discharge Type of Clinical Trial: Stratified, balanced, randomized clinical trial; no blinding RS Hypotheses: Not a study to detect response shift. Hypothesized that extent of nursing intervention (complexity and QoL domains targeted) leads to increased likelihood of achieving improvement in relevant outcomes (those targeted by the intervention) Sample: 190 persons returning home directly from acute-care hospital following first/recurrent stroke with need for health-care supervision post-discharge due to low function, comorbidity, or isolation. Nurse case-manager group n = 96, usual care group n = 94 RS Methods: Binary indicator for each of 7 QoL domains (change of 0.5 SD); GEE to estimate odds ratio of improvement; multiple imputation used for missing change scores RS Findings: NA
Gandhi et al. [37]	Main Research Question: To investigate the influence of explanatory and confounding variables on HRQOL among hypertensive Coronary Artery Disease patients by taking into account the issues of measurement bias and response shift in measurement Drug/Intervention: Antihypertensive treatment with either a verapamil SR- or atenolol-based strategy to achieve blood pressure control Type of Clinical Trial: 2 armed trail RS Hypotheses: The phenomenon of RS in HRQOL might have been caused by clinical variables such as antihypertensive treatment strategies, change in systolic and diastolic pressure; which are considered as catalysts in RS theory Sample: 788 hypertensive Coronary Artery Disease patients taking anti-hypertensive drugs and have depressive symptoms. Not given how many in each arm RS Methods: Structural Equation Modeling RS Findings: Patients reporting worsened Physical Functioning and Role Physical scores can be explained by individuals undergoing the process of recalibration response shift over time, and patients recalibrated their perception of Physical Functioning over a 1 year period, which was attributed to gender
Nirenberg et al. [38]	Main Research Question: Whether the intervention itself may impact on the accuracy of self-report (trust, knowledge re target behavior) Drug/Intervention: Community Service vs Motivational Interviewing Type of Clinical Trial: RCT; blinding not mentioned RS Hypotheses: No Sample: 478 people court-referred for high-risk driving and/or alcohol or other drug charges, Community service n = 160, Motivational Interviewing n = 318 RS Methods: Compared ratings with rankings and defined levels of agreement RS Findings: Ratings reflect what matters and rankings reflect what matters most; ratings are stable over time, rankings are more variable
Sajobi et al. [39]	Main Research Question: Is there reprioritization of HRQOL in patients treated surgically and/or medically for temporal lobe epilepsy? Drug/Intervention: Surgery vs. medical treatment Type of Clinical Trial: RCT, unblinded RS Hypotheses: Various domains of HRQOL will demonstrate RS over 1-year in patients with TLE randomized to either surgical or medical treatment Sample: 80 TLE patients randomized to surgical or medical treatment. Surgical group n = 40, medical group n = 40 RS Methods: Lix, Sajobi, et al. Relative Importance Method for reprioritization RS Findings: Evidence for reprioritization in social function and seizure domains of QOLEI
Mollerup and Johansen [33]	Main Research Question: To determine whether response shift effects are present in PROs of hand eczema severity Drug/Intervention: Nursing counseling vs. usual care Type of Clinical Trial: Secondary analysis of RCT; unblinded RS Hypotheses: RS is present in patient assessment of eczema severity Sample: 224/306 patients with hand eczema enrolled in a trial of nursing support. (no. per arm not provided) RS Methods: Defined presence of RS as the proportion of patients who endorsed lower VAS for worst-ever at 6 months compared to baseline RS Findings: Referred to "downwardly adjusting their assessment of VAS worst."
Machuca et al. [48]	Main Research Question: To describe response-shift patterns in people with dentine hypersensitivity using Classification and Regression Trees (CART) and to explore the convergent validity of CRT with the then-test and ideals approaches Drug/Intervention: 8-week clinical trial of mouthwashes for dentine hypersensitivity Type of Clinical Trial: Secondary analysis of 4-arm RCT; unblinded RS Hypotheses: No Sample: 75 general population participants with dentine hypersensitivity RS Methods: CART, the then-test and ideals analysis were conducted with the screening and week-8 assessments to investigate recalibration
Hoerger et al. [49]	Main Research Question: Does educating patients about evidence from the Early Palliative Care Study would increase preferences for palliative care? Drug/Intervention: Intervention participants received a web-based summary of the Early Palliative Care Study; controls received no intervention Type of Clinical Trial: Not stated but after submitting their data, participants were given more information about the study, links to educational and mental health resources, and controls were provided with access to the intervention materials. Thus, it was probably single blind and then unblinded for participants after data were collected RS Hypotheses: Yes: educating patients would lead them to be more likely to prefer palliative care Sample: 598 patients with prostate, breast, lung, colon/rectal, skin, and other cancer diagnoses. Intervention n = 309 and control n = 289 RS Methods: ANCOVA model testing for intervention effects (IV) on preferences (DV) after adjusting for demographic and clinical factors (covariates) Reliable change index focuses on proportion of individuals experiencing change, rather than mean changes at the group level. The RCI accounts for whether observed changes exceed what would be expected given the test–retest reliability of the measure and sampling error RS Findings: The intervention had a favorable impact on participants’ preferences for emotional, cognitive, and behavioral aspects of palliative care. Preferences for palliative care increased by about 3/4 SD among intervention participants. The intervention effect remained in sensitivity analyses using a more conservative outcome indicator
Murata et al. [40]	Main Research Question: Investigated the quantification of the response shift–adjusted treatment effect on quality-of-life (QOL) data in a randomized controlled trial Drug/Intervention: Taxane versus S-1 Type of Clinical Trial: open-label, noninferiority, phase 3 RS Hypotheses: Implied that there would be RS effects in RCT data Sample: 290 metastatic breast cancer patients: Taxane n = 139 and S-1 n = 151 RS Methods: Structural equation modeling techniques in addition to quantifying the “true” treatment effect. Measurement invariances in the values of the common factor loadings, intercepts, and residual variances between before treatment and at the 3-, 6-, and 12-month visits were considered the response shift effects RS Findings: In the Taxane group, we observed positive recalibration effects for role functioning and positive reprioritization and negative recalibration effects for emotional functioning. In the S-1 group, we observed positive reprioritization and negative recalibration effects for emotional functioning and positive reprioritization effects for social functioning "Decomposition of the changes between before treatment and the 3-month visit showed that most of the positive changes in emotional functioning were related to reprioritization response shifts in both treatment groups. This result reflected patient relief or calmness from psychological depression after a diagnosis of cancer metastasis or progression and a priority shift from a patient’s own health conditions to other things, for example, usual activities, while the negative impact of diagnosis would be almost temporary. In contrast, the negative changes in physical, role, and cognitive functioning in the Taxane group were related to nonresponse shift effects, including the true change from the treatment. This finding is interpreted as a reflection of the adverse effects of Taxane treatment such as fatigue and sensory neuropathy." "More accurate decision making requires further improvement in the evaluation reliability of observed HRQOL data during clinical trials through detailed analyses of the response shift effects…Careful consideration regarding the response shift effect in their study design would be required to observe a reliable conclusion without bias."
Sanders et al. [41]	Main Research Question: Whether lack of difference between treatments in Goal-Concordant Care was due to methodological challenges with how GCC measured Drug/Intervention: Serious Illness Care Program Type of Clinical Trial: Cluster-RCT; blinding not mentioned RS Hypotheses: None Sample: 203 patients with advanced cancer. Unclear how many in each group RS Methods: Compared ratings with rankings and defined levels of agreement RS Findings: Ratings reflect what matters and rankings reflect what matters most; ratings are stable over time, rankings are more variable
Schwartz et al. [43]	Main Research Question: To investigate possible response-shift effects in a recent clinical trial testing a new treatment (the drug Eculizumab in preventing relapse) for Neuromyelitis Optica Spectrum Disorder (NMOSD) Drug/Intervention: Eculizumab vs. placebo Type of Clinical Trial: A randomized, double-blind trial RS Hypotheses: Treatment arm and then relapse status were hypothesized ‘catalysts’ of response shift Sample: 143 patients with NMOSD: Eculizumab n = 95, placebo n = 48 RS Methods: The analysis began by testing an omnibus response-shift hypothesis and then implemented a series of random-effects models to elucidate specific response-shift effects. A series of random-effects models are: 1) testing group differences in patterns of emphasis (characterizing a recalibration response shift), 2) testing group differences over time in patterns of emphasis (characterizing reprioritization response shift), and 3) predicting each QOL domain from catalyst group after adjusting for other QOL domains (characterizing reconceptualization response shift) RS Findings: In the omnibus test, the standard QOL model captured substantially less well the experience of placebo as compared to Eculizumab group. Treat-related recalibration and reconceptualization response-shift effects were detected. Detected relapse-related response shifts included recalibration, reprioritization, and reconceptualization
Schwartz et al. [11]	Main Research Question: Given treatment and relapse-related response shift effects in VAS among people with NMOSD who participated in a clinical trial comparing Eculizumab to Placebo, this study aimed to “back-translate” the VAS into the MCS/PCS scores that would have been observed if response shift had not been present Drug/Intervention: Eculizumab vs. placebo Type of Clinical Trial: A randomized, double-blind trial RS Hypotheses: Response-shift effects were obfuscating treatment arm differences in mental health Sample: 143 patients with NMOSD: Eculizumab n = 95, placebo n = 48 RS Methods: The analysis sought to estimate MCS and PCS scores at baseline and at end of study by Treatment Arm, with and without response-shift effects. To translate MCS and PCS scores from the predicted value of VAS with and without response-shift effects, the classical-test theory method of equipercentile ranking was used to quate scores. A serial of random intercept models were implemented: Model 1 included fixed effects representing recalibration and reprioritization response-shift effects (i.e., group-by-MCS (or PCS) and group-by-MCS (or PCS)-by-time) and estimated the VAS scores with response effects. Model 2 estimated VAS scores without response shift, presumed that VAS ratings were attributable solely to MCS/PCS and treatment, but not to recalibration and reprioritization in the interaction terms (i.e., obtained VAS scores that removed variance related to recalibration and reprioritization response shifts). Model 3: took the estimated VAS without response-shift effects (predicted VAS value from model (2) and then adding the residual from model 1 to account for idiosyncratic variabilities under response shift RS Findings: Eculizumab patients’ MCS and PCS scores that include response-shift effects have a more truncated range, which generally makes them look better off than scores that remove response-shift effects. In contrast, Placebo patients’ crosswalks for both MCS and PCS exhibit similar ranges and similar linked scores whether including or excluding response-shift effects. Also, Placebo only and were more prominent at extremes of the MCS score distribution
Verdam et al. [45]	Main Research Question: To gain insight into relevance of RS in psychological health intervention research Drug/Intervention: Various psychological interventions Type of Clinical Trial: 3 RCTs: (1) internet-delivered CBT for insomnia in general population (n = 52/arm); (2) meaning-centered group psychotherapy for cancer survivors (n = 170 total or 85 per arm); (3) internet-based CBT for diabetes patients with depression (n = 255 total or 130 and 125 per arm) RS Hypotheses: That response shifts occur in the primary self-reported outcomes, is induced by psychological treatment, will occur only in the intervention groups, and not in the control groups. That the detected response shifts are clinically meaningful and substantially impact the assessment of change. That is, when response shift is taken into account, the treatment effects will be different (reduced or amplified). Moreover, that clinical and background variables are predictive of response shift Sample: 104 insomniacs; 170 cancer survivors; 255 depressed diabetics RS Methods: Structural Equation Modeling RS Findings: RS in both arms but did not affect trial results. RS may provide insight into differential treatment effectiveness
Schwartz et al. et al. [44]	Main Research Question: Do patients treated with ravulizumab or eculizumab for 26 weeks experience adaptation in their assessment of HRQOL compared to the general population? Drug/Intervention: 26 week treatment with either ravulizumab or eculizumab Type of Clinical Trial: Secondary analysis of two Phase 3 open-label studies evaluation non-inferiority of Ravulizumab compared to Eculizumab; general population data came from a cross-sectional sample from 11 European countries RS Hypotheses: Patients whose condition is well managed will evidence RS effects Sample: Patients with PNH treated with one of two agents participating in one of two phase 3 clinical trials (Trial 301, n = 246; Trial 302, n = 195); General population (n = 15,386) RS Methods: Multivariate analysis of covariance; Estimation of effect sizes; Logistic regression framework to test for differential item functioning RS Findings: Recalibration (uniform DIF over time in physical function, role function, emotional function, fatigue, and pain) and Reprioritization (non-uniform DIF over time in emotional, fatigue, and pain); Ravulizumab showed larger effect sizes; no treatment DIF but did uncover group DIF and DIF over time

*RS* response shift

## Abbreviations

EMA: European Medicines Agency
FDA: Federal Drug Administration
QOL: Quality-of-life
